# Modeling Prostate Cancer Treatment Responses in the Organoid Era: 3D Environment Impacts Drug Testing

**DOI:** 10.3390/biom11111572

**Published:** 2021-10-22

**Authors:** Annelies Van Hemelryk, Lisanne Mout, Sigrun Erkens-Schulze, Pim J. French, Wytske M. van Weerden, Martin E. van Royen

**Affiliations:** 1 Department of Urology, Erasmus University Medical Center, Dr. Molewaterplein 40, 3015 GD Rotterdam, The Netherlands; a.vanhemelryk@erasmusmc.nl (A.V.H.); l.mout@erasmusmc.nl (L.M.); s.erkens-schulze@erasmusmc.nl (S.E.-S.); 2 Department of Medical Oncology, Erasmus University Medical Center, Dr. Molewaterplein 40, 3015 GD Rotterdam, The Netherlands; 3 Cancer Treatment Screening Facility, Erasmus University Medical Center, Dr. Molewaterplein 40, 3015 GD Rotterdam, The Netherlands; p.french@erasmusmc.nl; 4 Department of Neurology, Erasmus University Medical Center, Dr. Molewaterplein 40, 3015 GD Rotterdam, The Netherlands; 5 Department of Pathology, Erasmus University Medical Center, Dr. Molewaterplein 40, 3015 GD Rotterdam, The Netherlands; m.vanroyen@erasmusmc.nl

**Keywords:** prostate cancer, CRPC, organoid, 3D cell culture, preclinical models, live-cell imaging, scaffold, drug testing, cell viability

## Abstract

Organoid-based studies have revolutionized in vitro preclinical research and hold great promise for the cancer research field, including prostate cancer (PCa). However, experimental variability in organoid drug testing complicates reproducibility. For example, we observed PCa organoids to be less affected by cabazitaxel, abiraterone and enzalutamide as compared to corresponding single cells prior to organoid assembly. We hypothesized that three-dimensional (3D) organoid organization and the use of various 3D scaffolds impact treatment efficacy. Live-cell imaging of androgen-induced androgen receptor (AR) nuclear translocation and taxane-induced tubulin stabilization was used to investigate the impact of 3D scaffolds, spatial organoid distribution and organoid size on treatment effect. Scaffolds delayed AR translocation and tubulin stabilization, with Matrigel causing a more pronounced delay than synthetic hydrogel as well as incomplete tubulin stabilization. Drug effect was further attenuated the more centrally organoids were located in the scaffold dome. Moreover, cells in the organoid core revealed a delayed treatment effect compared to cells in the organoid periphery, underscoring the impact of organoid size. These findings indicate that analysis of organoid drug responses needs careful interpretation and requires dedicated read-outs with consideration of underlying technical aspects.

## 1. Introduction

In the last two decades, three-dimensional (3D) organoid culture techniques have been revolutionizing in vitro preclinical research [1]. A remarkable collection of epithelial organoids has been established from healthy, diseased and cancerous tissues, both of human and animal origin. These 3D organoids fill the experimental gap between cell lines and complex animal models, while accurately preserving patient-specific phenotypic and genetic characteristics [2,3,4]. Encouraging results from recent studies using patient-derived tumor organoids for personalized drug response profiling highlight the exceptional potential of these near-patient preclinical models in oncology research [5,6,7,8]. Additionally, in prostate cancer (PCa) research, organoids have been shown to be promising tools for preclinical drug testing and for studying emerging treatment resistant phenotypes [9,10,11,12,13,14,15,16]. 

However, organoid-based studies currently lack standardized culture methods and drug testing procedures, introducing important methodological variability that hampers experimental reproducibility and validity. This is even more complicated by the absence of an exact definition for the term organoid, making it a motley collection of 3D culture systems [17,18] (including suspension cultures [12], hanging drop methods [19,20], scaffold-based models [9,10,13], etc.), for which the terms spheroid and organoid are often used interchangeably [21]. Here, we adhere to the conventional definition of organoids being 3D self-organizing organotypic structures cultured in a 3D scaffold [4,21,22]. The scaffold provides structural support for organoid assembly, while reproducing key cell-matrix interactions [23]. Natural scaffolds such as Matrigel, Geltrex or Cultrex, all murine-derived basement membrane matrices, are ubiquitously used for organoid culturing and functional applications [2,3,24]. However their variable and poorly defined composition, along with their animal origin, impede reproducibility regarding organoid differentiation and organoid responses to chemical compounds [23,25,26]. Recently emerged synthetic hydrogels, on the other hand, provide a controlled environment with well-defined physical, mechanical and biological properties [23,25]. Besides the organoid culture method applied, current drug testing protocols also vary in timing of treatment initiation, seeding of single organoid cells versus assembled organoids, organoid size, drug dosing, drug exposure time and read-outs [2,7,8,12,13,14,24,27,28,29]. Many of these factors have been implied to affect drug testing results [8,30,31,32].

We, therefore, aimed to explore the impact of 3D organoid organization and 3D environment on treatment efficacy using PCa organoids. We found these organoids to be less affected by standard of care chemotherapeutics and anti-androgens as compared to the same cells treated prior to organoid assembly. Confocal time-lapse live-cell imaging of taxane-induced tubulin stabilization and androgen-induced nuclear androgen receptor (AR) translocation revealed a time-dependent impact of 3D scaffolds, spatial organoid distribution and organoid size on drug effect.

## 2. Materials and Methods

### 2.1. Culture and Viability Assays of Metastatic Prostate Cancer Organoid Lines

The AR-negative (AR-) MSK-PCa1 and AR-positive (AR+) MSK-PCa2 organoid lines were kindly provided by Dr. Wouter Karthaus and Prof. Dr. Jack Schalken from the Radboud University Medical Center. Organoids were cultured and passaged as described previously [9,33], with the addition of synthetic androgen R1881 (1 nM; Sigma-Aldrich, Saint Louis, Missouri, USA, cat. no. R0908) for MSK-PCa2 organoids. Viability assays were conducted in pre-warmed 96-well plates (Costar, Corning, New York, NY, USA, cat. no. 3595) at a plating density of 2500 MSK-PCa1 or 5000 MSK-PCa2 organoid cells in 8 µL Matrigel domes (Corning, cat. no. 356231) per well. Single cells or organoids were incubated for 7 days with a dose range of docetaxel, cabazitaxel (0.01–10 nM; provided by Sanofi, Paris, France), abiraterone (0.03–30 µM; provided by Sanofi) or enzalutamide (0.03–30 µM; Axon Medchem, Groningen, the Netherlands, cat. no. 1613). Viability was measured with CellTiter-Glo 3D according to the manufacturer’s protocol (Promega, Madison, WI, USA, cat. no. G9681).

### 2.2. Live Cell Imaging of Androgen-Induced Nuclear AR Translocation

The human AR+ PCa cell line PC346C [34] was stably transduced to express enhanced green fluorescent protein (EGFP) labelled AR as described previously [35]. PC346C EGFP-AR cells were maintained in steroid-stripped prostate growth medium (PGM-DCC) [36] without corticosteroids and R1881 to omit AR activation. Cells were seeded at a density of 2000–3500 cells per well in black 384-well CellCarrier Ultra plates suited for high content imaging (Perkin Elmer, Hamburg, Germany, cat. no. 6057302) and were allowed to settle for 48 h. Prior to live cell imaging, cells were incubated with Hoechst 33342 (2 µg/mL; Invitrogen, Thermo Fisher Scientific, cat. no. H3570) for 2 h. Medium was removed and cell monolayers were covered either with 20 µL Matrigel or 20 µL synthetic polyisocyanopeptide (PIC) hydrogel (Noviogel-P1K, Noviocell, Sopachem, Ede, The Netherlands; stock solution containing a 35.5: 64.5 ratio of Noviogel and medium). Gels were allowed to solidify for 10 min at 37 °C and subsequently topped with warm medium. Replicate wells without gel were included as controls. For 3D experiments, PC346C EGFP-AR cells were plated in 8 µL Matrigel or Noviogel domes in black 96-well CellCarrier Ultra plates (Perkin Elmer, cat. no. 6055302) and assembled into organoids within a week. Before imaging, organoids were incubated with Hoechst 33342 (4 µg/mL) for 3 h. Confocal time-lapse imaging was performed with the Opera Phenix High Content Screening System (Perkin Elmer) equipped with a 40× water immersion objective and a 16 bit sCMOS 4 Megapixel camera. Baseline images were acquired before adding R1881 to culture medium (at a final concentration of 10 nM), followed by single-plane time-lapse images every 15 min for 8 h in monolayer experiments and every 30 min for 5 h in organoid experiments. Hoechst 33342 and EGFP-AR were excited with 405 and 488 nm solid state lasers and detected at 435–480 and 500–550 nm wavelength ranges, respectively. Images were processed using the Harmony analysis software (version 4.9, Perkin Elmer). Organoids were segmented based on the combination of Hoechst 33342 and EGFP signals. Nuclear and perinuclear cytoplasmic regions of cells in both 2D monolayers and 3D organoids were segmented based on the nuclear Hoechst 33342 staining and the incomplete translocated EGFP-AR signals. At each time point, the relative nuclear EGFP-AR translocation was determined as nuclear EGFP signal intensity/(nuclear EGFP signal intensity + cytoplasmic EGFP signal intensity) after reduction of background, with 0 representing complete cytoplasmic and 1 complete nuclear translocation [35]. When relevant, the relative localization of organoids within the Matrigel dome was calculated for each organoid as follows: $distance\;from\;the\;center=\surd \left(x\;position^{2}+y\;position^{2}\right)$. Distribution of organoids was assessed and organoid cells were grouped in 9 bins of ~250 µm. The average relative AR nuclear translocation was calculated per bin for each time point.

### 2.3. Live Cell Imaging of Taxane-Induced Tubulin Stabilization

Through long-term propagation of PC346C cells in steroid-stripped and androgen-deprived culture conditions, the castration-resistant PC346C-DCC-K subline was generated [36,37]. Cells were subsequently transfected to overexpress enhanced yellow fluorescent protein (EYFP) labelled beta-tubulin (β-tubulin) [38]. For live cell imaging experiments, 10^4^ cells were seeded per well in 384-well plates and allowed to settle for 72 h. Cells were overlaid with Matrigel or Noviogel as described above and replicate wells without gel were included as controls. Baseline confocal images were obtained pretreatment, after which cabazitaxel was added to culture medium (at a final concentration of 3 nM) to induce tubulin stabilization. Images were acquired at intervals of 30 min for a total time frame of 18 h and analyzed using the Harmony software. EYFP-β-tubulin was detected at 488 nm excitation and 500–550 nm emission wavelength. Total cell area was segmented based on the EYFP-β-tubulin signals. Within this area, tubulin-stabilizing effects of cabazitaxel were quantified based on tubulin bundle formation using the Haralick contrast of the EYFP signal in the total cell area, as previously published [38,39]. For 3D experiments, PC346C-DCC-K EYFP-β-tubulin cells were plated in 8 µL Matrigel domes in 96-well plates and allowed to assemble into organoids. Tubulin stabilization in these organoid experiments was only qualitatively assessed, as the 3D organization of tubulin interfered with the quantitative analysis described above.

### 2.4. Statistical Analysis

GraphPad Prism (version 9, GraphPad Software, San Diego, CA, USA) and R software (version 4.1.0 [40]) were used for data visualization and statistical analyses. Dose-response curves were generated using the nonlinear regression curve fit method (4 parameters) in Prism. Nonlinear curve fitting was used to determine t_1/2_ of nuclear AR translocation and tubulin stabilization, followed by pairwise unpaired *t* tests (two-tailed) between separate groups. The level of statistical significance was set at 0.05. Bonferroni correction was applied in case of multiple testing.

## 3. Results

### 3.1. 3D Organoid Structure Attenuates Sensitivity to Taxane-Based Chemotherapy and Anti-Androgen Treatment

**Figure 1:**
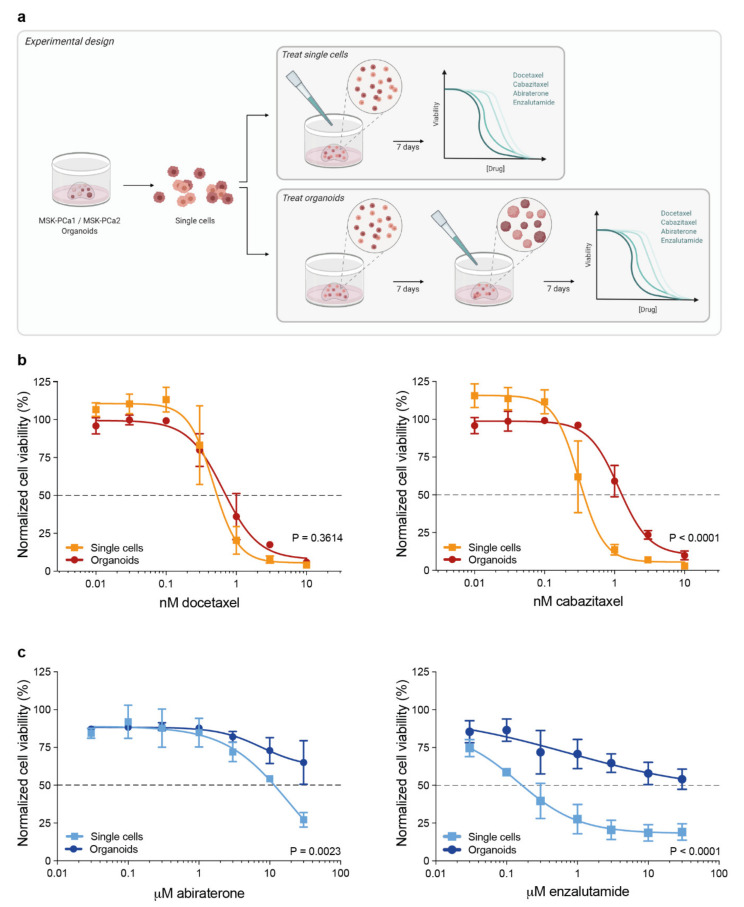
Dose-response experiments of prostate cancer (PCa) organoids and complementary three-dimensional (3D) cultures of dissociated single organoid cells. (**a**) Schematic representation of the experimental design. (**b**) Dose-response curves of androgen receptor (AR)-negative MSK-PCa1 organoids exposed to taxanes (mean +/− SEM of four independent experiments with three technical replicates per dose). Cell viability was normalized to vehicle (ethanol) controls. (**c**) Dose-response curves of AR-positive MSK-PCa2 organoids exposed to anti-androgens (mean +/− SEM of three independent experiments with three technical replicates per dose). Cell viability was normalized to vehicle (DMSO) controls.

To evaluate the effect of the 3D cellular organization of organoids on their responses to contemporary therapies in metastatic PCa, we exposed single organoid cells and assembled organoids to dose-ranges of docetaxel, cabazitaxel, abiraterone and enzalutamide (Figure 1a). MSK-PCa1 organoid cells were embedded in Matrigel and incubated with taxanes either immediately after seeding of dissociated cells, or after seven days when organoids were formed. No significant difference in drug response was observed between both groups after incubation with docetaxel. However, exposing assembled organoids to cabazitaxel elicited a significant shift in the dose-response curve compared to immediate exposure after seeding (*p* < 0.0001, Figure 1b), indicating a reduced cabazitaxel sensitivity due to the 3D organoid organization. In a similar fashion, the AR+ MSK-PCa2 organoid line was exposed to anti-androgen treatment directly after seeding of single cells or when organoids were established. This triggered a prominent reshaping of dose-response curves, revealing an impaired drug effect of abiraterone and enzalutamide in cultures of established organoids (Figure 1c).

### 3.2. Extracellular Matrix Affects Androgen-Induced AR Translocation and Chemotherapy Effectiveness

**Figure 2:**
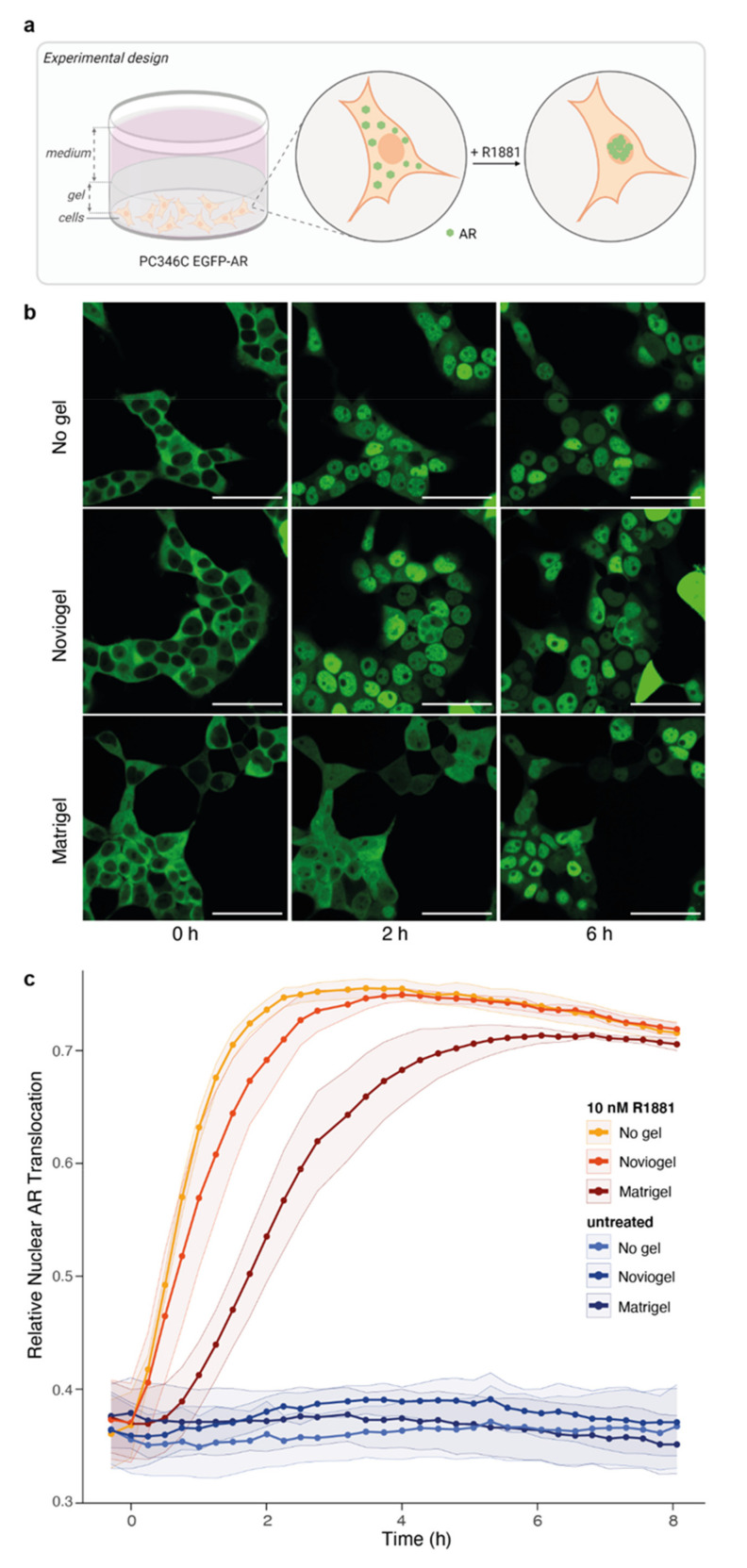
Impact of scaffolds on androgen-induced AR translocation. (**a**) Schematic representation of experimental design. EGFP-AR: enhanced green fluorescent protein (EGFP) labelled AR. (**b**) Representative images of PC346C EGFP-AR monolayers not covered with scaffold (no gel, top panel), covered with synthetic scaffold (Noviogel, middle panel) or covered with Matrigel (bottom panel) at 0, 2 or 6 h after the addition of synthetic androgen (10 nM R1881). Scale bars represent 50 µm. (**c**) Image-based quantification of nuclear AR translocation in cells not covered with scaffold (no gel, yellow), covered with Noviogel (orange) or with Matrigel (red) following exposure to 10 nM R1881. Untreated wells without addition of R1881 (shades of blue) were included as controls. Data represents the mean +/򲈒 SEM of three individual experiments performed with four technical replicates per condition. Time to reach t_1/2_ of control wells (no gel) was minimally delayed by Noviogel (*p* = 0.3632) and significantly delayed by Matrigel (*p* = 0.0089).

A major difference between 2D cell lines and 3D organoid models is the presence of a scaffold supporting organoid assembly and mimicking extracellular matrix. We hypothesized that these scaffolds, in which organoids are embedded, could delay drug availability and hence drug effect on the embedded cells. In order to assess this scaffold-mediated delay of drug effect without interference of the 3D organoid structure (as compared to our data above), we seeded a 2D cell monolayer and covered these EGFP-labeled AR expressing cells with Matrigel or Noviogel (Figure 2a). Upon addition of synthetic androgen (10 nM R1881), we observed a rapid translocation of the AR to the cell nucleus in control wells without a scaffold layer. However, in cells covered with the synthetic scaffold Noviogel a delay in nuclear AR translocation of approximately 1 h was observed. In cells covered with the natural scaffold Matrigel, this delay was even more pronounced, to more than 3 h before reaching plateau levels (Figure 2b,c).

**Figure 3:**
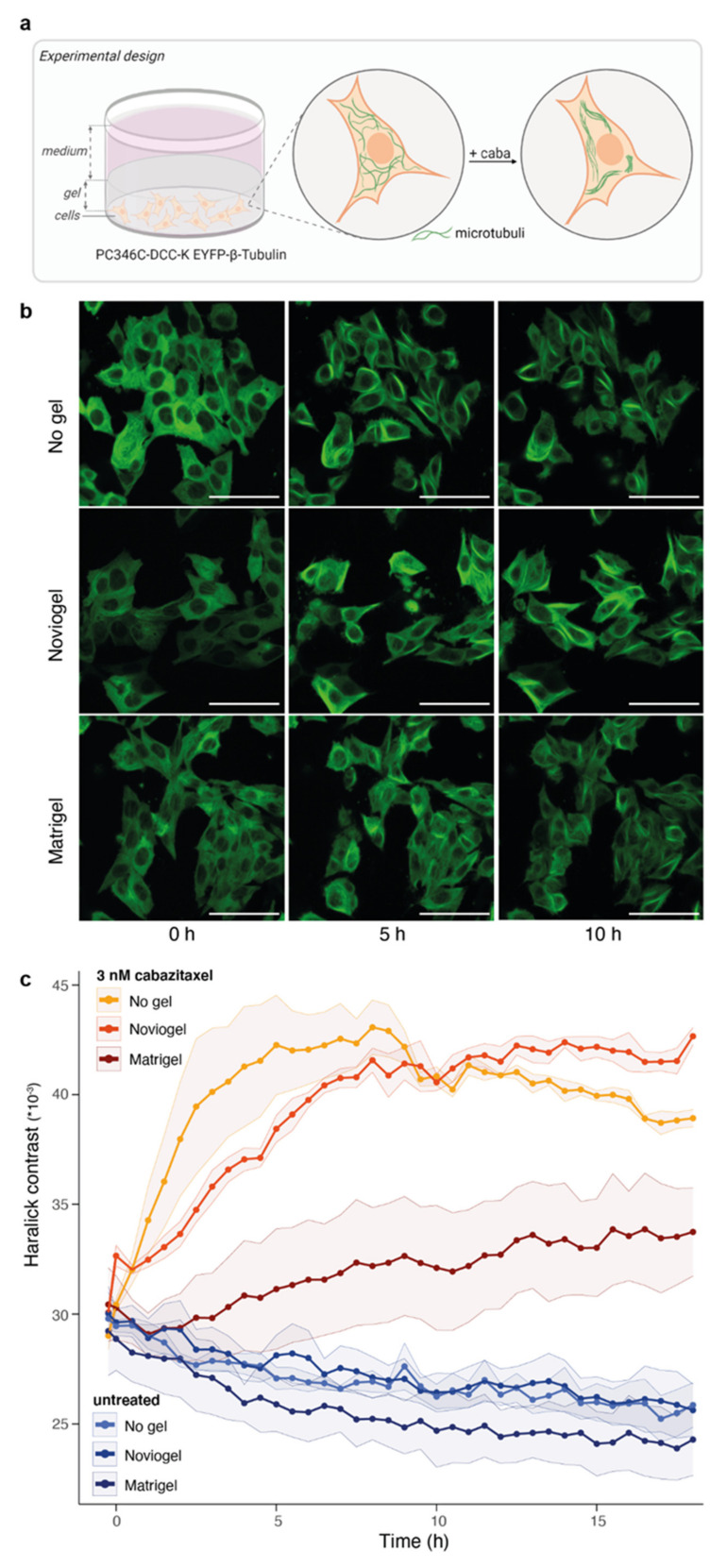
Impact of scaffolds on taxane-induced tubulin stabilization. (**a**) Schematic representation of experimental design. EYFP-β-Tubulin: enhanced green fluorescent protein labelled beta-tubulin; caba: cabazitaxel (**b**) Representative images of PC346C-DCC-K cells expressing EYFP-β-Tubulin that were not covered with scaffold (no gel, top panel), covered with synthetic scaffold (Noviogel, middle panel) or with Matrigel (bottom panel) at 0, 5 or 10 h after the addition of 3 nM cabazitaxel. Scale bars represent 50 µm. (**c**) Image-based quantification of cabazitaxel-induced tubulin stabilization, using Haralick contrast values, in cells not covered with scaffold (no gel, yellow), covered with Noviogel (orange) or with Matrigel (red) following exposure to 3 nM cabazitaxel. Untreated wells without addition of cabazitaxel (shades of blue) were included as controls. Gradual decrease in Haralick contrast values over time is likely to be caused by bleaching. Data represents the mean +/− SEM of four individual experiments performed with four technical replicates per condition. Time to reach t_1/2_ of control wells (no gel) was non-significantly delayed by Noviogel (*p* = 0.1513) and was not attained in the Matrigel condition.

As an alternative read-out, we used taxane-induced tubulin stabilization to interrogate the potential effect of the selected scaffolds on cabazitaxel effectiveness. PC346C-DCC-K cells expressing EYFP-labelled β-tubulin were seeded as a monolayer and covered with scaffold (Figure 3a). As previously observed [38,41,42], cabazitaxel induced rapid tubulin stabilization in control wells without scaffold, as measured by Haralick contrast [39] (Figure 3b,c). The presence of Noviogel delayed tubulin stabilization by approximately 3 h, but final Haralick contrast values plateaued at the same level as in control wells. In contrast, Matrigel strongly deferred tubulin stabilization and Haralick contrast values did not even reach saturation within the time frame of 18 h, suggesting an incomplete level of tubulin stabilization.

### 3.3. Spatial Distribution of Organoids Impacts Compound Effectivity

**Figure 4:**
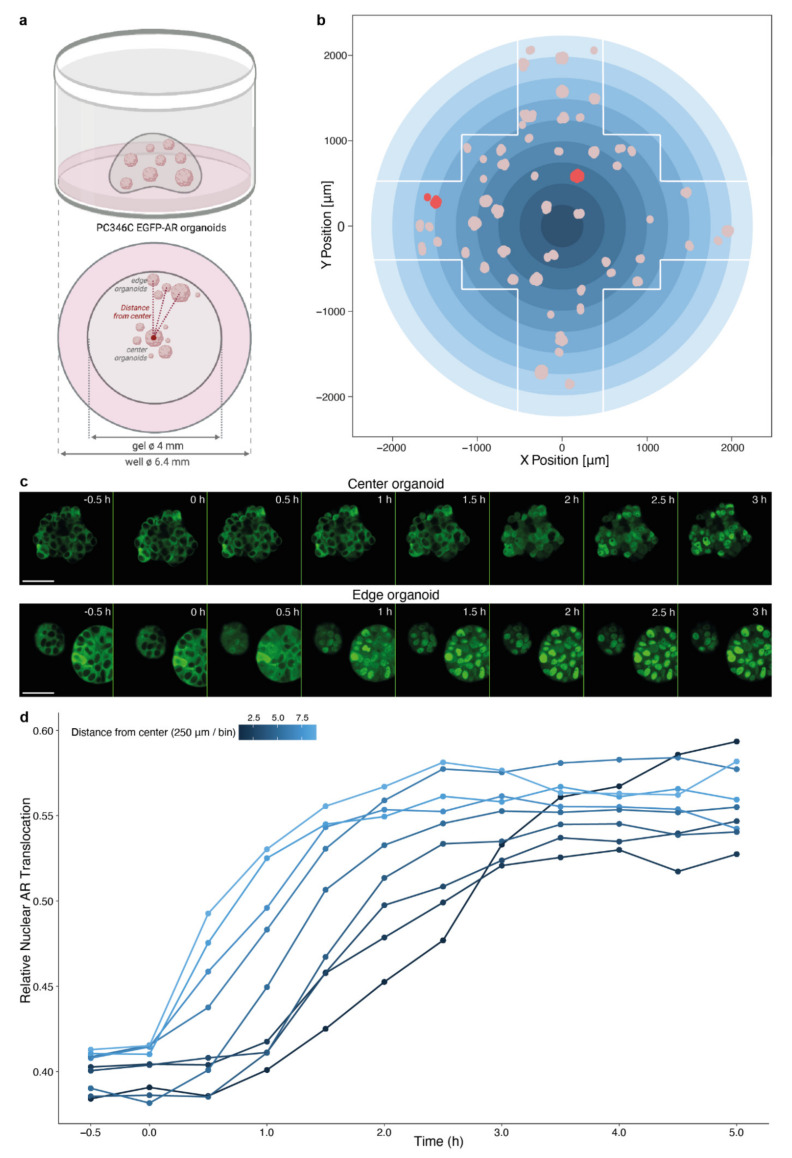
Impact of spatial organoid distribution within a Matrigel dome on androgen-induced AR translocation. (**a**) Schematic representation of experimental design. (**b**) Illustration of organoid distribution within a single imaged well, based on the X position and Y position. White lines delineate the imaging field. Pink dots depict organoids, red dots are the organoids shown as example in (**c**). Gradient blue circle represents bins applied in (**d**). (**c**) Representative images of androgen-induced AR translocation in a PC346C EGFP-AR organoid located at the center (top panel) or edge (bottom panel) of the Matrigel dome following exposure to synthetic androgen (10 nM R1881). Time after exposure is indicated in the right upper corner of each image. Scale bars indicate 50 µm. (**d**) Image-based quantification of nuclear AR translocation in organoid cells grouped in nine bins of ~250 µm, based on the distance from the center of the well as depicted in (**b**). 10 nM R1881 was added to organoid medium at 0 h. Data points represent the average of four imaged wells.

Because of the prominent impairment of drug effect induced by a layer of Matrigel, we further aimed to evaluate the spatial impact of organoid localization within a Matrigel dome, as this represents a common experimental setting for organoid drug testing. Again, we used androgen-induced nuclear AR translocation as a readout, now in PC346C EGFP-AR organoids embedded in a Matrigel dome that was positioned in the center of the well. Spatial localization within the dome was determined for each imaged organoid and visualized based on distance from the center of the well (and thus Matrigel dome; Figure 4a,b). Following exposure to R1881 (10 nM), organoids more centrally in the dome demonstrated a delay in nuclear AR translocation as compared to organoids in the Matrigel dome periphery (Figure 4c,d). 

In a similar manner, tubulin dynamics were investigated in PC346C-DCC-K EYFP-β-tubulin organoids cultured in Matrigel. Although quantification of tubulin stabilization was not feasible in a 3D setting because of the 3D orientation of the stabilized tubulin bundles, qualitative assessment revealed a comparable impact of organoid position on cabazitaxel effectivity. This was reflected in both time of initiation and the apparent level of tubulin stabilization (Figure S1), as was seen for the 2D monolayer analysis in the Matrigel condition (Figure 3b,c). These data corroborate the effect of Matrigel in an experimental organoid setting and imply a potential impact when assessing overall organoid drug responses.

### 3.4. Impact of Organoid Size on Drug Effectivity

**Figure 5:**
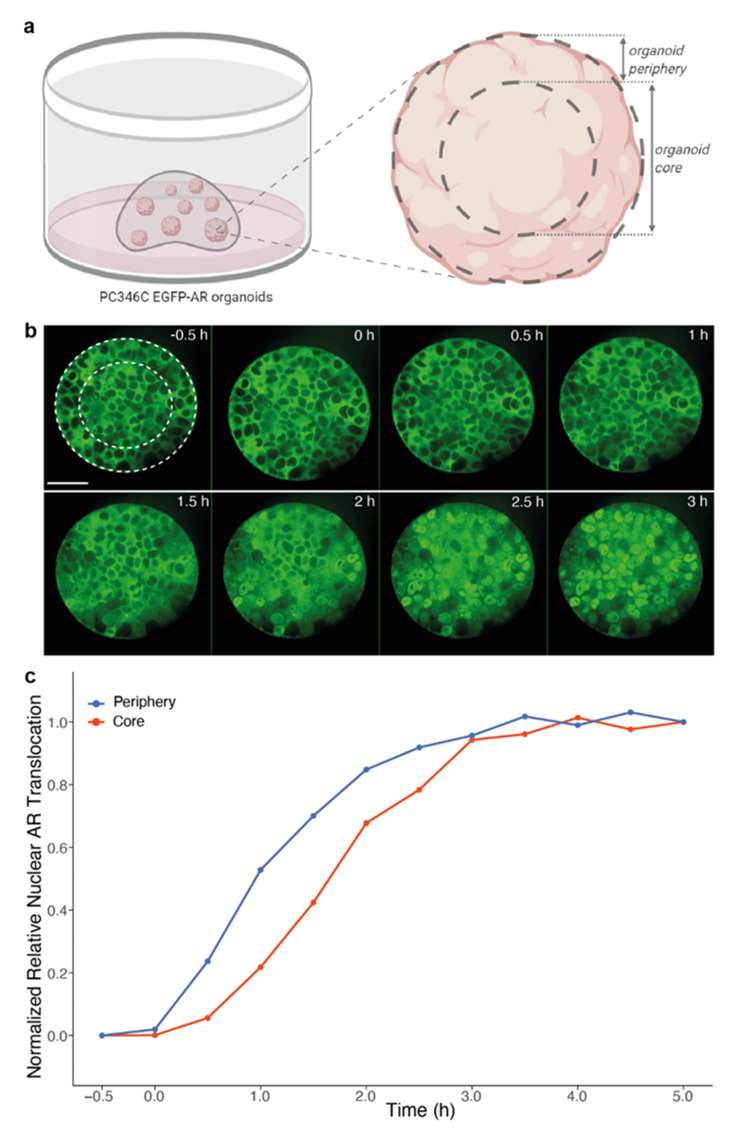
Impact of organoid size on androgen-induced AR translocation. (**a**) Schematic overview of segmentation within individual organoids. (**b**) Representative images of androgen-induced AR translocation within a single PC346C EGFP-AR organoid. Time after exposure to 10 nM R1881 is indicated in the right upper corner of each image. White dotted circles represent segmentation in organoid core versus organoid periphery. Scale bar indicates 50 µm. (**c**) Image-based quantification based on the segmentation within individual organoids depicted in (**a**) and (**b**). Nuclei located inside an outer rim of 100 pixels from the organoid circumference were allocated to the organoid periphery, nuclei located inwards of this organoid periphery (thus > 100 pixels from the organoid circumference) were allocated to the organoid core. Curves represent the normalized relative nuclear AR translocation in cells from large organoids (with a cross-sectional area > 8000 µm^2^) from five imaged wells (*n* = 47 organoids). Curves were normalized to allow direct comparison of the dynamics of AR translocation between the often dimmer, low-contrast organoid core and the brighter, high-contrast organoid periphery (*p* < 0.0001 at 1 h; *p* < 0.0001 at 2 h, unpaired *t* test, two-tailed).

Finally, to verify the impact of the 3D organoid structure on drug effectivity as concluded from Figure 1, we compared androgen-induced nuclear AR translocation in cells along the organoid periphery to cells located in the organoid core (Figure 5a). To minimize the scaffold-mediated delay and the effect of organoid location within the dome demonstrated in Figure 2, Noviogel was used for embedding PC346C EGFP-AR organoids. In large individual organoids (selected based on a cross-sectional area >8000 µm^2^), cells in the organoid periphery more rapidly manifested nuclear AR translocation than cells in the organoid core; while no differences were observed in small organoids (Figure 5b,c). These findings indicate that organoid size is an additional variable that affects drug activity in organoid-based studies.

## 4. Discussion

Organoids are highly promising preclinical models that provide perspectives for patient-tailored treatment guidance based on matching organoid drug sensitivities [8,43]. However, results from a first large prospective intervention trial on colorectal cancer patients demonstrated only a limited value of patient-derived organoids to guide individualized treatment selection [44]. Technical complexities associated with these 3D models, combined with the lack of methodological standards for organoid-based drug testing, could well be complicating experimental reproducibility and clinical implementation. We observed a significantly impaired drug effectivity in our PCa organoids compared to their complementary 3D cultures of dissociated organoid cells, thus suggesting a reduced drug sensitivity caused by the 3D structure of organoids. Similar observations were made in studies comparing drug sensitivities of cancer cell lines cultured as either 2D monolayers or 3D aggregates [31,45,46,47]. We hypothesized underlying mechanistic factors to be diverse and interrelated, such as organoid drug penetration, intratumoral drug accumulation, spatial drug distribution or the occurrence of a drug concentration gradient across a single organoid, as was also previously observed in cell line-derived spheroids [47] and ex vivo cultures of tumor fragments [48]. Using PCa organoids as a model, we aimed to further investigate the impact of the 3D organoid organization and 3D culture environment on treatment efficacy in time when performing organoid-based drug tests.

A fundamental feature of organoid culture technology is the use of 3D scaffolds to recapitulate key cell-matrix interactions [21,23]. We hypothesized that these 3D scaffolds might form a physical barrier for drug availability, as also suggested by others [12]. We therefore investigated the impact of Matrigel (the most commonly used natural scaffold in organoid cultures) and Noviogel (a synthetic PIC hydrogel) on the dynamics of drug efficacy. Even in 2D conditions, where the effect of the hydrogel is isolated from potential biological changes due to 3D cell culture, a layer of each of the scaffolds delayed AR translocation and tubulin stabilization, with Matrigel inducing a more pronounced treatment impairment than Noviogel. Within a time span of 18 h, Matrigel covered cells demonstrated incomplete tubulin stabilization after treatment with taxanes, which was not observed for the synthetic hydrogel. The observed difference in drug activity between both scaffolds might be explained by Matrigel’s complex and poorly defined biochemical and mechanical properties, as well as its introduction of murine growth factors and xenogenic contaminants into the culture environment [23], potentially affecting drug diffusion and effectivity. As organoids are conventionally embedded within a scaffold dome, these scaffold-mediated delays might translate into spatially divergent treatment effects between individual organoids. Endorsing this assumption, we indeed noted a delayed AR translocation for organoids located centrally in a Matrigel dome as compared to organoids at the dome periphery. Similarly, Shin et al. [26] reported on a considerable diffusion limitation of the morphogen Wnt3a into the Matrigel dome, creating a concentration gradient that caused Wnt3a depletion for organoids in the core of the Matrigel dome and induced spatial heterogeneity in organoid morphology and proliferation [26]. This phenomenon might equally occur for nutrients and chemical compounds, as implied by our results. Extending this reasoning to the 3D structure of the organoids themselves, we hypothesized that a drug concentration gradient might arise across a single organoid. Our observation of delayed androgen-induced nuclear AR translocation in cells located in the organoid core as compared to cells in the organoid periphery supports this hypothesis and is also in concordance with previous work that detected preferential accumulation of drugs in cells in the outer spheroid layer [47]. Such gradients might explain decreased drug sensitivity with increasing spheroid or organoid size, as also detected by Edmondson et al. [49]. Appropriate adjustment of drug concentration and drug exposure time might resolve these issues [47,48]. 

Although the AR translocation and tubulin stabilization readouts are direct and well-known effects of the treatments with hormones and taxanes respectively [35,41,42], these readouts are only indirect indications for drug availability or drug efficacy and further research is warranted to directly quantify drug concentration within the scaffold dome and within individual organoids (e.g., by the use of fluorescently labelled drugs). Another limitation of this study is that we compared only two different scaffolds; Matrigel was chosen for its ubiquitous use in the organoid field, while the synthetic PIC hydrogel, Noviogel, was chosen for its well-defined composition and thermo-sensitive properties. Importantly, this study focused on the mechanical factors impacting drug responses; however, cell responses and cell fate in 3D cultures could well be impacted by other underlying, cell biological, mechanisms. When comparing to their 2D counterparts, 3D cultures have demonstrated alterations in epigenetic regulation [50], gene expression [51], protein expression [49], post-translational modifications [30], metabolism [52] and differentiation status [51]. Upregulation of genes involved in cell-cell adhesion, gap junctions proteins and extracellular matrix interactions is correlated with chemo-resistance in 3D models [53], confirming that not only technical aspects presented in this study, but also important biological aspects impact drug-related research when shifting towards 3D cell culture models.

In conclusion, our findings indicate that analysis of organoid drug responses needs careful interpretation and consideration of underlying technical aspects. Although organoid cultures better represent the complex 3D physiology of solid tumors, clinical translation of organoid-based drug sensitivities necessitates methodological standards for organoid drug testing, with meticulous attention to technical details such as culture method, organoid size, drug concentration, drug exposure time and read-outs.

## Data Availability

The datasets generated and/or analyzed during the current study, as well as the PC346C cell line and its variants used in this study, are available from the corresponding author upon reasonable request.

## Supplementary Materials

The following are available online at https://www.mdpi.com/article/10.3390/biom11111572/s1, Figure S1: Impact of spatial organoid distribution within a Matrigel dome on taxane-induced tubulin stabilization.
